# Exercise as a multitarget therapy: modulating myokines, neurotrophins, and inflammation in Parkinson’s disease

**DOI:** 10.3389/fnagi.2025.1580029

**Published:** 2025-08-26

**Authors:** Wei-Qi Li, Jia-Hua Yang, Lu-Lu Liu, Song-Tao Ding, Bin Yu, Lin Jiang, Ning Yan, Han-Deng Liu

**Affiliations:** ^1^Laboratory of Tissue and Cell Biology, Experimental Teaching and Management Center, Chongqing Medical University, Chongqing, China; ^2^College of First Clinical, Chongqing Medical University, Chongqing, China; ^3^Department of Neurology, University-Town Hospital of Chongqing Medical University, Chongqing, China; ^4^Key Laboratory of Major Brain Disease and Aging Research (Ministry of Education), Chongqing Medical University, Chongqing, China; ^5^Center for Neuroscience Research, Chongqing Medical University, Chongqing, China

**Keywords:** Parkinson’s disease, exercise, cytokines, irisin, BDNF, TNF-α

## Abstract

Parkinson’s disease is a progressive neurodegenerative disorder characterized by degeneration of dopaminergic neurons, leading to significant motor and non-motor symptoms. Recent studies emphasize that exercise is a beneficial intervention, not only helping to decrease the risk of developing of Parkinson’s disease but also alleviating existing symptoms. This review investigates the mechanisms by which exercise influences myokines, neurotrophic factors, growth factors, and inflammation-related factors to promote neuronal survival and plasticity in Parkinson’s disease. Despite promising findings, the specific molecular pathways through which exercise exerts neuroprotective effects remain largely unexplored, and individual variability in disease progression necessitates personalized exercise interventions tailored to each patient’s needs. Furthermore, for patients unable to engage in physical activity, exploring alternative therapies that mimic exercise to achieve neuroprotective effects is crucial. In conclusion, this review highlights the need for further research to elucidate the molecular mechanisms of exercise-induced neuroprotection and to establish effective individualized exercise programs, ultimately improving the management of Parkinson’s disease.

## 1 Introduction

Parkinson’s disease (PD) represents a neurodegenerative condition primarily affecting dopaminergic neurons situated within the substantia nigra region of the midbrain. This disorder unfolds through a gradual clinical progression, encompassing a spectrum of symptoms that include premotor, non-motor, and motor manifestations. Collectively, these symptoms profoundly impair patients’ quality of life and contribute to substantial healthcare expenditures (Leite Silva et al., 2023). Simultaneously, caregivers of those diagnosed with PD are under tremendous stress (Macchi et al., 2020). The primary movement-related signs characteristic of PD encompass bradykinesia, muscle tonus, quiescent tremor, and postural instability (Ahmad et al., 2023). Although the etiology of PD continues to be enigmatic, contemporary research indicates that the development of Lewy bodies (Shahmoradian et al., 2019), neuroinflammation (Tansey et al., 2022), mitochondrial damage (Eldeeb et al., 2022), and intestinal flora dysbiosis (Zheng et al., 2021), and other factors that may be closely related to PD.

Age constitutes the primary risk factor associated with the onset of PD (Tolosa et al., 2021). With the ongoing enhancement of survival rates within the elderly population, a corresponding rise has been observed in the total count of individuals diagnosed with PD, a trend that is anticipated to continue. This situation is described by some as a pandemic (Dorsey et al., 2018). There is often a long delay (10 years on average) between the first manifestation of symptoms and the time of diagnosis in people with PD, due to the fact that PD is challenging to identify in its initial phase (Gaenslen et al., 2011). Initial manifestations of the condition include constipation (the most prevalent indication), the occurrence of dream activity occurring during the rapid eye movement (REM) sleep stage (suggestive of REM sleep-behavioral dysfunction), diminished sense of smell, unilateral and indistinct shoulder discomfort, or depressive symptoms (Armstrong and Okun, 2020). None of these early manifestations are sufficient to establish a designation of PD by themselves, as each of them can also occur as a part of many other diseases. As a result, the disease is typically diagnosed late in the course of complete neuronal degeneration, and treatment is always delayed (Lotankar et al., 2017). Taking early preventive measures becomes an effective way to reduce the incidence of PD. It has been increasingly demonstrated that a certain amount of physical activity can prevent neurodegeneration, which is associated with PD (Ritz and Paul, 2022). Under certain assumptions, introducing vigorous physical activity in a population could prevent 14.5% of current PD cases (Ben-Shlomo et al., 2024).

Exercise not only decreases the risk of developing of PD, it is also an effective treatment. For motor symptoms in PD patients, effective therapeutic interventions such as Tai Chi, yoga, or traditional balance exercises can optimize balance and mobility (Zhang T. et al., 2023). Motor deficits of rat models simulating PD are ameliorated after prolonged treadmill routine interventions. Following physical exercise, there is a reduction in oxidative stress levels and an enhancement in mitochondrial function observed in rat models (Landry et al., 2022; Liu L. et al., 2024; Mu et al., 2022; Tung et al., 2024). Expiratory muscle training has the potential to markedly diminish the overall seriousness of dysphagia in individuals suffering from PD (Claus et al., 2021). Meanwhile, physical activity can also affect Parkinson’s-related non-motor manifestations and may represent a viable therapeutic approach for alleviating fatigue among those with PD (Folkerts et al., 2023). Yoga also has a considerable impact on anxiety and depression among patients with PD (Kwok et al., 2019). Furthermore, dance interventions offer both motor and non-motor benefits for Parkinson’s disease (PD). Within the sensorimotor domain, therapeutic effects predominantly manifest in balance improvement, gait enhancement, reduction of motor symptom severity, and increased functional mobility among individuals with PD (Bek et al., 2020). Through structured dance participation, persons with PD experience not only neuromotor improvements but also opportunities for self-actualization and social reintegration, with measurable benefits in combating disease-related stigma and enhancing perceived wellbeing (Bognar et al., 2017). It is shown that physical activity also affects the pathologic manifestations associated with PD in related studies. Aerobic exercise (but not stretching) leads to enhanced connectivity function between the anterior shell region and the sensorimotor cortex area relative to the posterior shell nucleus (Johansson et al., 2022), and exercise on a treadmill alleviates the propagation of α-syn (Dutta et al., 2022). Nonetheless, it is of paramount importance to acknowledge that effective exercise is a significant strategy for reducing the risk of PD and assisting in the treatment of PD as well as reducing its mortality rate, but only with appropriate intensity and duration of exercise (Bhadra et al., 2023; Stein et al., 2021; Tsukita et al., 2022; Zhang X. et al., 2022). Engaging in excessive physical exercise can result in bodily tiredness, and the equilibrium of oxidative/antioxidant systems in the body can be disrupted, which in turn causes damage to the central nervous system (CNS) (Bai et al., 2023).

The favorable outcome of exercise in PD is undisputed, but the mechanisms of action are unclear. These mechanisms may involve neuronal survival and plasticity, neurogenesis, epigenetic modifications, angiogenesis, autophagy, etc (Sujkowski et al., 2022). In numerous studies, the impacts of physical activities on the body’s metabolism have garnered extensive discussion, particularly regarding its promotion or inhibition of the production of related cytokines. This paper will delve into the promoting influence of physical exercise on the production and secretion of cytokines, as well as the intrinsic mechanisms of these factors in alleviating symptoms of PD. We will primarily concentrate on myokines, neurotrophic factors, growth factors, and inflammation-related factors. The intrinsic mechanisms of various exercise-induced factors hold significant research importance, as they could prepare for a novel treatment for PD patients who already experience motor impairments and those who are unable to engage in physical activity.

## 2 Myokine

Myokines are a class of peptide molecules produced and secreted by skeletal muscles during exercise and are considered one of the key mechanisms underlying the neuroprotective effects of physical activity. These factors exert beneficial effects through various regulatory pathways, including promoting cell survival, neurogenesis, modulation of neuroinflammation, maintenance of proteostasis, resistance to oxidative stress, and protein modification. Recent studies on exercise-induced myokines have opened new avenues for the treatment of neurodegenerative diseases (Lee et al., 2021). To date, only a few myokines have been systematically investigated for their roles in such disorders. In this section, we focus on two well-studied myokines, irisin and cathepsin B (CTSB), and their effects on neurodegenerative diseases. However, further studies are urgently needed to identify more myokines and comprehensively evaluate their therapeutic potential in neurodegenerative conditions.

### 2.1 Irisin

Irisin is a muscle-derived myokine first identified as an exercise-responsive hormone, acting as a crucial molecular bridge between physical activity and systemic health. Its secretion is induced by physical exercise through the upregulation of *PGC1-*α, which activates the transcription of the *fibronectin type II domain-containing protein 5 (FNDC5)* gene. *FNDC5* encodes a type I transmembrane protein that undergoes proteolytic cleavage to release a soluble peptide, irisin. Circulating irisin levels are influenced by exercise intensity and correlate with *FNDC5* expression in skeletal muscle (Boström et al., 2012; Huh et al., 2012). Although controversy once surrounded its expression in humans—due to the non-canonical ATA start codon in the human *FNDC5* gene and the low specificity of commercial antibodies (Albrecht et al., 2015; Raschke et al., 2013)—several mass spectrometry-based studies have successfully validated the presence of irisin in human blood and cerebrospinal fluid (Jedrychowski et al., 2015; Lee et al., 2014; Ruan et al., 2018). Beyond its metabolic roles, irisin has attracted attention for its protective effects across multiple organ systems, including improving liver function (Polyzos et al., 2014), enhancing systemic glucose metabolism (Perakakis et al., 2017), maintaining musculoskeletal homeostasis (Zhao et al., 2024), and suppressing cancer progression (Pinkowska et al., 2021). Notably, irisin also shows promising potential in neurological disorders. *FNDC5* is expressed in the brain and involved in neural differentiation and maturation (Baghi et al., 2021). In PD models, *FNDC5* expression is downregulated during the acute phase but elevated in the chronic phase, likely reflecting a compensatory response (Tsai et al., 2021). Irisin can cross the blood-brain barrier, and increased peripheral levels are associated with elevated central concentrations. In the brain, irisin regulates neurogenesis, synaptic plasticity, inflammation, and cognitive function (Islam et al., 2021).

Once secreted, irisin circulates and targets tissues to exert its effects. The irisin secretion cycle targets tissues and exerts its physiological effects through the binding to cellular aV family integrin complex receptors, especially aVb5 phosphorylation of the 397 site of Focal Adhesion Kinase (FAK) after ligand binding. It is worth noting that this signal transduction has high specificity. In HEK293T cells expressing integrin aVb5, irisin treatment significantly enhanced the phosphorylation level of FAK; However, similar effects were not observed in cells expressing integrin aVb3 (Kim et al., 2018). The present research found that the interaction between irisin and the aVb5 receptor is facilitated by the motility-inducing molecule Heat Shock Protein 90α (Hsp90α). The activation of the aVb5 receptor demonstrates a strong attraction toward irisin (Mu et al., 2023). It was confirmed that uncut *FNDC5* could directly promote the secretion of BDNF and enhance synaptic plasticity by binding to integrin aVb5 receptor on the surface of hippocampal neurons in MPTP induced chronic PD mouse models. Additionally, *FNDC5* promotes dopaminergic connectivity from the substantia nigra to the hippocampus by mediating the interaction between integrin aVb5 in hippocampal neurons and CD90 molecules on the dopaminergic terminal membrane. It is notable that *FNDC5* directly binds to the receptor without splicing, while irisin binds to the receptor after splicing (Tang C. et al., 2023). Nonetheless, the complete identification of irisin receptors remains an unresolved challenge in the field (Pignataro et al., 2021), while the intracellular signaling pathways of irisin have been largely elucidated. Irisin primarily exerts its biological functions through mitogen-activated protein kinase (MAPK) signaling pathways, with involvement in processes such as neural differentiation, osteoblast proliferation, and osteogenic differentiation (Rabiee et al., 2020). In addition, irisin mediates additional physiological effects through alternative signaling pathways. For instance, irisin can induce white adipose tissue browning via p38 and ERK signaling pathways (Zhang et al., 2014), The ERK1/2 MAPK signaling cascade mediates endothelial cell proliferation processes (Liu et al., 2017). Irisin demonstrates anti-metastatic activity in lung cancer by reversing EMT processes through PI3K/AKT-dependent inhibition of Snail signaling (Shao et al., 2017).

Currently, there are relevant studies that have demonstrated that dietary changes are unable to influence the standards of irisin within the body (Ko et al., 2016); exercise may be the only current method to increase circulating irisin in the body. For example, related studies have shown that despite similar body weights, baseline levels of irisin were observed to exhibit higher levels in individuals who have undergone training compared to those who have not, suggesting that exercise exerts a direct effect on the elevation of irisin (Algul et al., 2017). Performed on different exercise modalities, compared to endurance training alone as well as in comparison to exercise regimens that incorporate both resistance and endurance components, resistance training resulted in a more significant enhancement of the irisin response (Kim H. J. et al., 2015). Furthermore, the intensity of exercise also has an effect on irisin production. In mouse experiments, male subjects were randomly allocated to a sedentary control group (CO), a free-contact running wheel group (RW), and a running group (TM); furthermore, the findings indicated that the concentrations of irisin were increased in the mice belonging to the TM group immediately after acute exercise. However, in the RW group, the *PGC1-*α*/FNDC5*/irisin pathway did not respond significantly to mild exercise (Brenmoehl et al., 2014). The experiment demonstrates that moderate to high-intensity exercise increases the expression of *PGC1-*α, which in turn elevates the levels of *FNDC5*, subsequently increasing circulating irisin levels (Benedini et al., 2017). However, there are also studies that offer a different view on whether exercise can increase irisin levels: chronic exercise training brings about a notable diminution in the levels of circulating irisin (Qiu et al., 2015), while acute strength training lasting up to 30 min did not cause a significant elevation of serum irisin levels (Pekkala et al., 2013). Although some studies also mentioned a decrease in irisin after exercise, most of the studies on irisin still showed an increase after exercise (Table 1), which could be due to differences in assay method or time of day.

**TABLE 1:** Comparative analysis of exercise interventions and changes in irisin levels.

References	Model	Exercise intervention	Exercise duration and frequency	Sample type and changes	Detection method
Lin et al., 2024	Wistar rats	Treadmill exercise, 35 min/time	8 weeks, 35 min/time, 5 times/week	Soleus muscle ↑ (*p* < 0.05) Compared with no-exercise	Western blot
Cosio et al., 2024	Human	40-m sprints	10 times, 3-min rest pause between each repetition	Blood ↑ (*p* < 0.001) Compared with pre-exercise	Kit against mass spectrometry
Dupuis et al., 2024	Sprague–Dawley rats	Sensorimotor restriction	28 days, 16 h/day	Blood ↑ (*p* < 0.05) Compared with the control	ELISA
Koltun et al., 2024	Human	Resistance and interval training program	12 weeks, 60–90 min/time, 3 times/week	Blood ↑ (*p* < 0.05) Compared with pre-exercise	ELISA
Jeong et al., 2024	Human	Resistance exercise	4 weeks, 70 min/time, 4 times/week	Blood ↑ (*p* < 0.05) Compared with pre-exercise	ELISA
Shang et al., 2024	Sprague–Dawley rats	Treadmill physical training	10 m/min, 10 min/d, 5 days/week	Blood ↑ (*p* < 0.05) Compared with pre-exercise	ELISA
Guazzarini et al., 2024	Human	Tai Chi	6 months, 2 times/week	Blood ↑ (*p* = 0.05) Compared with the control	ELISA
Tommasini et al., 2024	Human	Incremental cycling exercise		Blood ↑ (*P* < 0.001) Compared with pre-exercise	ELISA
Kim et al., 2023	Human	A high-intensity interval training session on a cycle ergometer	44 min, 1 time	Blood ↑ (*p* > 0.05) Compared with pre-exercise	ELISA
Izawa et al., 2024	SAMP8 rats	Aerobic exercise	16 weeks, 30 min/day, eight sets were conducted with 1-min intervals, 3 times/week	Hippocampal tissue ↑ (*p* < 0.001) Compared with the control	ELISA

**FIGURE 1:**
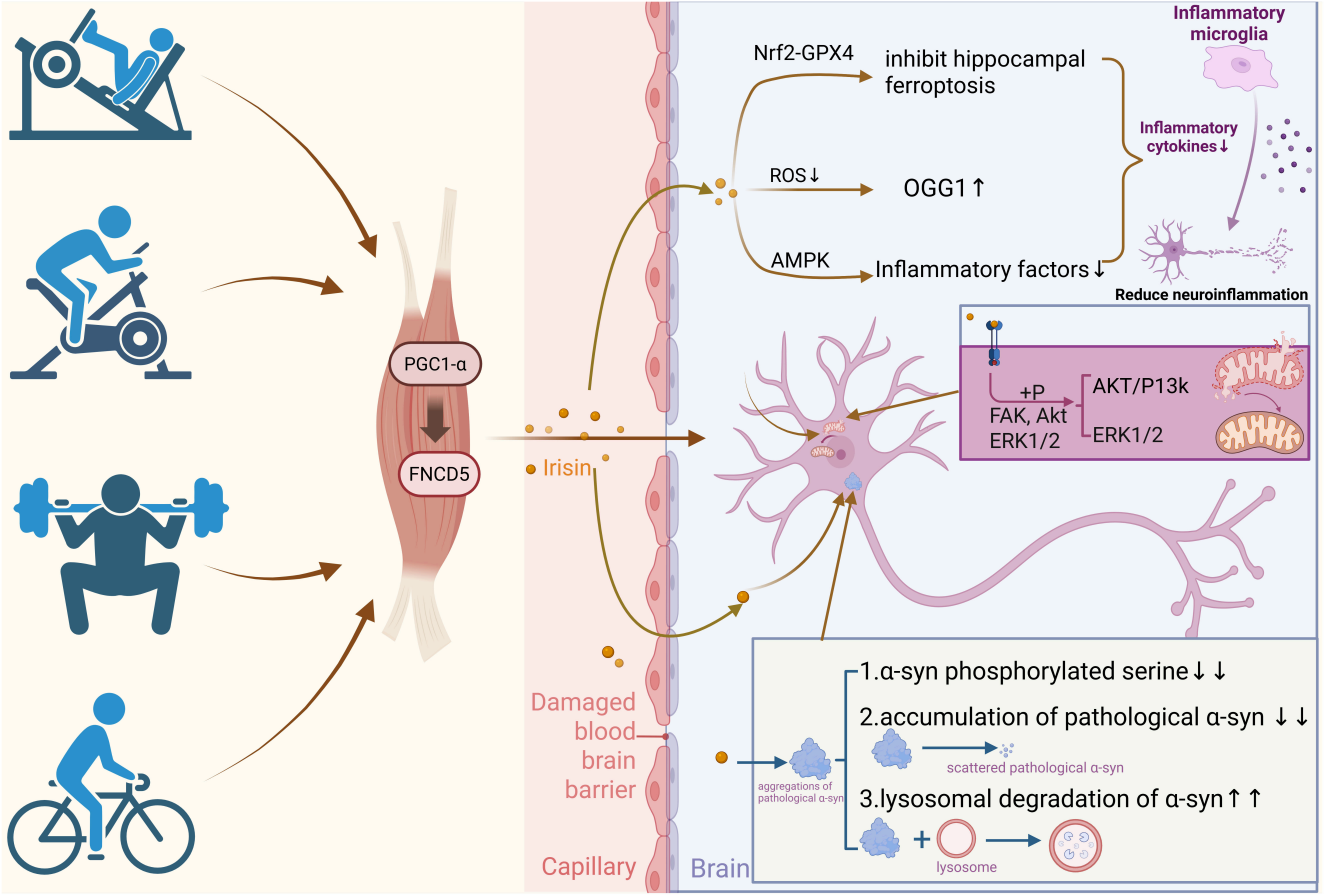
Exercise boosts irisin and helps PD treatment. When the human body engages in exercise, muscle cells are stimulated to secrete *PGC1-*α, which further promotes the expression of FNCD5. The irisin released by FNCD5 can cross the damaged blood–brain barrier and enter the brain, triggering a series of protective mechanisms. In terms of inhibiting neuroinflammation, irisin activates the AMPK signaling pathway, decreases the level of ROS (reactive oxygen species), and increases the expression of OGG1 (8–oxoguanine DNA glycosylase 1). Meanwhile, it activates the Nrf2–GPX4 pathway. Together, they inhibit ferroptosis. In addition, the levels of inflammatory factors decrease, and the AKT/P13k and ERK1/2 signaling pathways are also activated to update mitochondrial function, thereby reducing neuroinflammation. In the aspect of regulating α-synuclein (α-syn) metabolism, irisin reduces the number of phosphorylated serine on α-syn, prevents the aggregation of pathological α-syn, and promotes its degradation by lysosomes, thus alleviating the pathological accumulation of α–syn in the brain.

Irisin hormone, a myokine, has a vital impact on several neurodegenerative disorders (Ali et al., 2024), and various studies have demonstrated that irisin effectively inhibits the pathologic changes in PD (Figure 1). Neurodegeneration, which is induced by pathological α-synuclein (α-syn), was prevented by irisin in the α-syn preformed fibers (PFF) mouse model of PD, thereby preventing the deterioration of dopaminergic neurons and the subsequent decrease of striatal dopamine (De Sousa, 2024). The intrinsic mechanisms can occur in at least three ways: (1) The coming into being of α-syn phosphorylated at serine 129 and the death of neuronal cells were diminished by irisin. And then irisin conferred neuroprotective effects against the neurotoxicity induced by α-synuclein. (2) The internalization and aggregation of pathological α-syn were reduced by irisin; therefore, irisin could achieve the effect of reducing its accumulation. (3) The efficiency of irisin in enhancing lysosomal degradation-mediated synaptophysin clearance effectively prevented the pathological transmission of α-syn. In this process, it was experimentally demonstrated that, 1 and 4 days after α-syn PFF treatment, irisin reversed 100% and 34.6% of the affected proteins, respectively. Notably, irisin treatment led to a significant upregulation of the ApoE protein (Kam et al., 2022). The ε 4 genotype of ApoE protein regulates α-Syn pathology in humans (Davis et al., 2020) and is linked to a heightened risk of dementia among patients with PD, while ApoE ε 2 may prevent the aggregation of α-syn and its link with neurodegenerative processes in synucleinopathies (Bras et al., 2014; Mata et al., 2014). Other studies have determined by immunoblotting that irisin rescues dopamine transporter protein (DAT)-positive and tyrosine hydroxylase (TH)-positive fibers, prevented the loss of dopamine neurons and lowering of striatal dopamine (Kam et al., 2022), and effectively promotes dopamine absorption in the contralateral striatum of the affected limb in PD patients (Shi et al., 2024). Iron death shares common features with PD pathophysiology (Mahalakshmi et al., 2020). It is reported that irisin can improve the inflammatory microenvironment by inhibiting hippocampal ferroptosis through the Nrf2/GPX4 signaling pathway, significantly decreasing the abundance of ROS, MDA, ASCL4, and ALOX12, and decreasing the progression of neuroinflammation (Wang J. et al., 2022). In the neurotoxicity-induced PD model, irisin does not directly target mitochondria after being transported to cells but activates the signaling pathway through binding to the αV integrin receptor to exert protective effects. Irisin activates the phosphorylation of FAK, Akt, and ERK1/2. Then the signaling processes mediated by PI3K-Akt and ERK1/2 are activated. Next, it refreshes mitochondrial function, restores mitochondrial morphology, and prevents apoptosis and oxidative stress, etc. Besides, irisin can also alleviate the mitochondrial damage through the MAPK signaling pathway. It is noteworthy that during irisin treatment, it bypasses the AP site (DNA damage abasic site) caused by ROS and restores OGG1, the main enzyme responsible for repairing 8-oxoG as well (Zhang X. et al., 2023). While simultaneously, irisin has the capability to stimulate the AMPK signaling pathway within the brain, mitigating the generation of inflammatory cytokines and alleviating neuroinflammation and oxidative stress (Zhang Q. et al., 2024). Various studies have suggested that irisin may play a role in different animal models of PD. However, current research on its underlying mechanisms remains incomplete, and further prospective studies are needed to identify the specific molecular pathways through which irisin counteracts the pathological features of PD.

### 2.2 CTSB

Within the lysosome, cleavage of the propeptide converts procathepsins into mature cathepsins. Cathepsins are integral to various lysosome-related processes, including protein degradation, metabolism, renewal, antigen presentation, apoptotic signaling, phagocytosis, and growth factor receptor recycling, particularly in autophagy (Turk et al., 2012). Therefore, dysregulated expression or activation of cathepsins is associated with a wide range of autoimmune diseases, phlogosis (Patel et al., 2018), and even neurodegenerative diseases (Stoka et al., 2016). CTSB that encodes cathepsin B is the most prevalent cysteine protease among all lysosomal proteases and is widely expressed (Yadati et al., 2020), especially in the CNS, where it is linked to neuronal function (Tran and Silver, 2021).

Exercise mediates the advantageous consequences of CTSB on brain function through various mechanisms. Running increases the expression of the CTSB gene in the hippocampus. Physical activities can lead to low oxygen level (Radak et al., 2013), which can enhance the quantities of CTSB in the brain (Yakovlev and Gulyaeva, 2015). The removal of neurodegenerative fragments (Devi and Kiran, 2004) and adult neurogenesis can be promoted by this elevation, a process related to memory operations (Abrous and Wojtowicz, 2015). The suppression of CTSB and cathepsin L (CTSL) reduces the synthesis of BDNF in the hippocampus (Bednarski et al., 1998). BDNF engages in the regulation of synaptic plasticity, the survival of cells, and differentiation (Chao et al., 2006).

Genetic differences in CTSB have been proven to be hazardous factors for PD (Pedersen, 2019). A cellular characteristic of PD is the cytoplasmic amassment of α-syn and amyloid formation. CTSB can proteolyze the core and amyloidogenic regions of α-syn, thereby preventing the taking shape of α-syn fibrils (McGlinchey and Lee, 2015). There may be crosstalk between α-syn aggregation and lysosomal-autophagy damage (Bellomo et al., 2020). When α-syn aggregates, the transport of CTSB to lysosomes is disrupted, resulting in that proteolytic activity of cathepsins decreased. Thus, promoting the transport of CTSB to lysosomes can increase their activity, facilitating efficient degradation of α-syn (Drobny et al., 2023). In summary, exercise-mediated elevation of CTSB demonstrates its benefits for PD and highlights the importance of CTSB in degrading α-syn, highlighting its latent as a treatment mark for PD.

## 3 Neurotrophic factors

Neurotrophic factors are a group of secreted proteins that support neurogenesis, promote synaptic plasticity, and prevent neuronal degeneration under both physiological and pathological conditions. They play a crucial role in the survival, maintenance, and regeneration of specific neuronal populations in the adult brain (Allen et al., 2013). Physical exercise has emerged as an effective stimulus for the expression of neurotrophic factors, thereby contributing to neuroprotection in neurodegenerative diseases (Wang and Holsinger, 2018). Key neurotrophic factors influenced by physical activity include brain-derived neurotrophic factor (BDNF) and glial cell line-derived neurotrophic factor (GDNF). In this section, we summarize the regulation of exercise-induced neurotrophic factors and their potential therapeutic roles in neurodegenerative diseases.

### 3.1 BDNF

**FIGURE 2:**
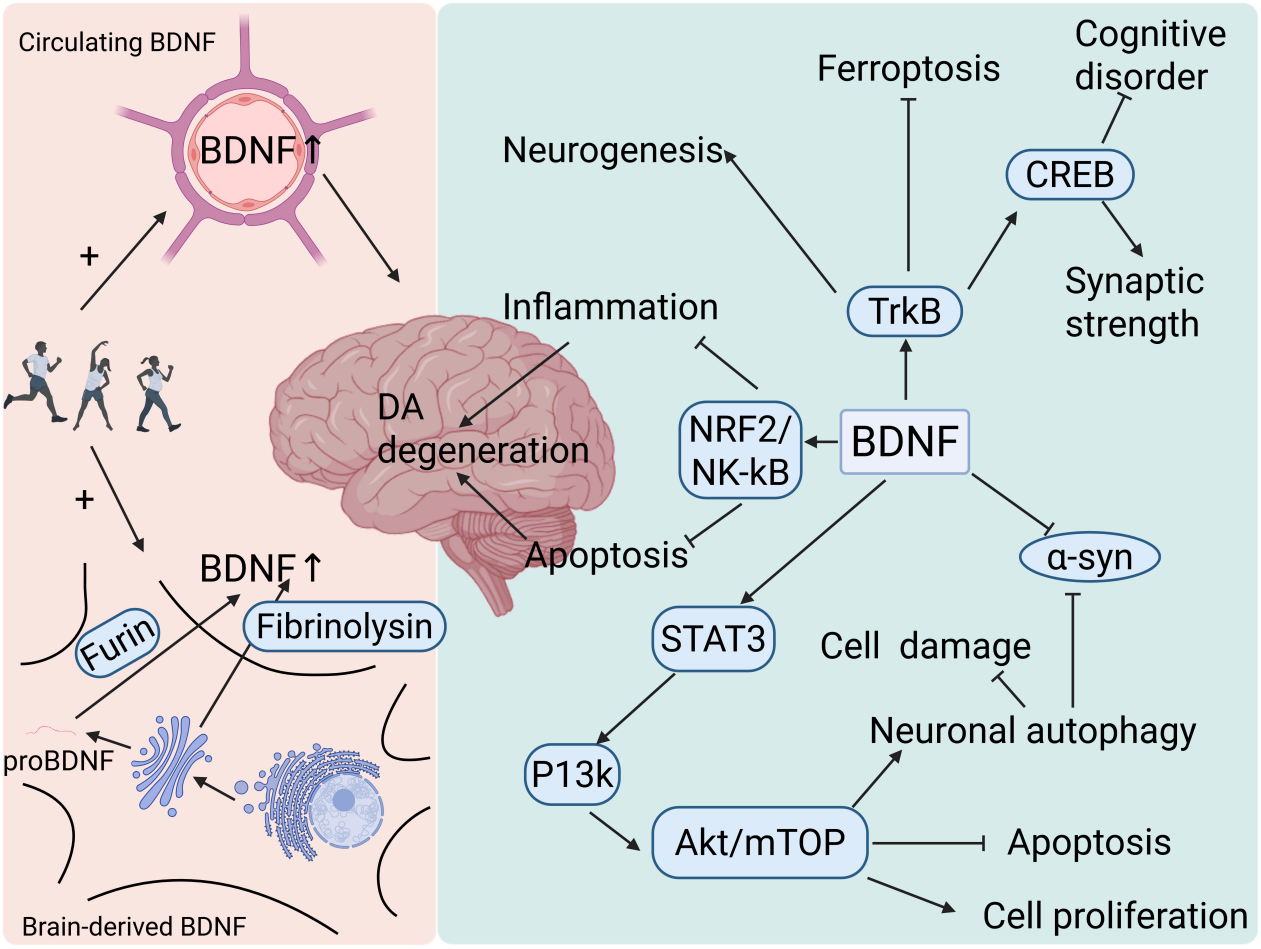
The link among exercise, Brain-derived neurotrophic factor (BDNF) and neuroprotection. Exercise enhances both central and peripheral expression of BDNF, providing neuroprotection through multiple pathways.

BDNF, it is regarded as the most plentiful neurotrophic factor in the brain of adults, with a role as a significant neurotrophic signal and neuromodulator (Bazzari and Bazzari, 2022). In the brain, BDNF is expressed by glutamatergic neurons (Andreska et al., 2014), glial cells (e.g., astrocytes isolated from the cortex and hippocampus but not from the striatum) (Clarke et al., 2018), and microglia (Parkhurst et al., 2013). It is found extensively in the CNS and PNS (peripheral nervous system), as well as in bone and cartilage tissues, and the highest concentration was located in the brain and cortex. The BDNF molecule consists of three structural domains: a signal peptide, a pre-structural domain, and a mature structural domain (Colucci-D’Amato et al., 2020). After entering the Golgi apparatus, the signal peptide is cleaved off. BDNF is first produced as the proBDNF precursor molecule; then, the proBDNF molecule can be cleaved intracellularly by furin or extracellularly by fibrinolytic enzymes or matrix metalloproteinases to form BDNF (Figure 2; Finan et al., 2018). Furin identified as the first proprotein convertase discovered in mammals (Thomas, 2002), cleaves proprotein substrates in the brain, including the precursor of BDNF (Zhang Y. et al., 2022). It plays a protective role in CNS injury in the brain mainly through binding to its specific receptor, the tropomyosin-related kinase receptor B (TrkB). The BDNF-activated TrkB signaling mechanism is critical for the survival of neurons, structural modifications, and plasticity (Wang et al., 2024).

Research has demonstrated that self-directed physical activity boosts the hippocampal expression of BDNF (Loprinzi et al., 2019), which in turn affects hippocampal neurogenesis (Fabel et al., 2009). Physical activity is known to increase circulating levels of BDNF (Figure 2; Knaepen et al., 2010; Szuhany et al., 2015), thereby improving brain function (Tuon et al., 2015; van Praag et al., 2005; Vilela et al., 2017). Circulating BDNF is present in cerebrospinal fluid and blood, and it has been demonstrated that circulating BDNF in plasma can cross the blood-brain barrier to some extent (Pan et al., 1998). The animal data also show a correlation between peripheral and central concentrations (Klein et al., 2011). Currently, the relationship between exercise and peripheral circulating BDNF has been well studied. A randomized controlled trial (RCT) consisting of 18 studies evaluated the effect of exercise intervention on plasma BDNF levels in patients with neurodegenerative diseases [i.e., multiple sclerosis, Parkinson’s disease, mild cognitive impairment (MCI), and Alzheimer’s disease] compared to a lack of exercise. Overall, the exercise intervention resulted in significantly higher plasma BDNF levels in neurodegenerative diseases (Ruiz-González et al., 2021). However, various forms of exercise impact BDNF in distinct ways. Several studies have indicated that after several weeks of resistance training, the amount of circulating BDNF rises and is independent of the type of resistance training (Yarrow et al., 2010). However, strength training does not appear effective in increasing BDNF (Huang et al., 2014). It has been established through research that aerobic exercises are effective in enhancing BDNF levels (Cassilhas et al., 2012). A meta-analysis including 910 participants (61.3% male, mean age: 42.2 years) revealed that blood concentrations of BDNF were dramatically increased by aerobic exercise rather than resistance exercise training (Dinoff et al., 2016). New research has once again found no major difference between aerobic and resistance training but rather a positive correlation with the duration of exercise (Dinoff et al., 2017). It is thought that BDNF levels increase after several weeks of regular repetitive training (Church et al., 2016). In addition, Nordic Walking has been proven by scholars to increase the level of BDNF. For patients with Parkinson’s disease of varied durations and severities, it represents a safe, feasible, and sustainable aerobic exercise modality (Harro et al., 2022). But it has also been suggested that interval sprint training is more likely to trigger BDNF than steady-state training at a moderate intensity and steady-state training at a high intensity (Reycraft et al., 2020). Although it is unclear which type of exercise maximizes BDNF, all types of exercise certainly have a function in the elevation of BDNF. The mechanisms behind this process by which exercise promotes BDNF production are not well studied, but some studies have suggested that it may be peripheral lactate levels that promote BDNF production (El Hayek et al., 2019), while others propose that exercise influences gut microbiota, indirectly promoting BDNF elevation (Molska et al., 2024). Additionally, some authors suggest that physical activity increases adrenergic (norepinephrine) or serotonergic (5-HT) signaling, thereby promoting BDNF expression (McMorris, 2016).

After binding to TrkB, BDNF activates intracellular signaling pathways, playing crucial roles in maintaining neuronal growth and survival. During the development of embryonic cells, BDNF-TrkB signaling promotes the development of cortical progenitor cells, leading to their maturation into neurons, i.e., neurogenesis (Fabel et al., 2009). It has been shown that BDNF-TrkB-CREB signaling in excitatory cortical neurons of layer V of the cortex mediates the control of slow wave activity (SWA) during sleep, which in turn affects synaptic strength (ElGrawani et al., 2024). Chen et al. (2023) suggested that hippocampal neurogenesis in neonatal HIBD (hypoxic-ischemic brain damage) rats can improve cognitive impairments by promoting the integrated signaling mechanism involving BDNF, its receptor TrkB, and CREB. Numerous clinical studies have found that PD patients have significantly reduced BDNF (Howells et al., 2000; Kang et al., 2017; Karakasis et al., 2011). Inhibiting BDNF expression in the substantia nigra (SN) leads to a PD-like phenotype in rats (Hernandez-Chan et al., 2015). In addition to this, BDNF variants can significantly heighten the possibility of LRRK2 induction among individuals who experienced PD at a certain age greater than 60 years, suggesting that there is a cumulative effect between the two genetic variants and that the simultaneous presence of both increases the likelihood of the development of PD (An et al., 2008), and variants at the LRRK2 locus are a significant genetic risk factor in the development of PD (Liu and LeRoith, 1999). The study of the LRRK2 gene began with the observation of an autosomal dominant inherited Parkinson’s disease case in a Japanese family in 1978. Subsequently, in 2002, the PARK8 locus was identified through linkage analysis, and the p.I2020T mutation was identified (Sosero and Gan-Or, 2023). Thereafter, other LRRK2 variants associated with Parkinson’s disease were identified (Ross et al., 2011). LRRK2 mainly causes harm to dopamine neurons through multiple mechanisms such as inflammatory response (Cook et al., 2017; Rui et al., 2018), lysosomal dysfunction (Eguchi et al., 2018), and mitochondrial dysfunction (Ludtmann et al., 2019), which suggests an important link between PD and BDNF. There are also many studies on the therapeutic effects of BDNF on PD. It has been demonstrated that BDNF in the dorsal striatum (dStr) increases after physical activities, which facilitates an augmentation in the secretion of dopamine in the dStr (Bastioli et al., 2022). Geng et al. (2023) showed a significant decrease in the levels of p-STAT3, p-PI3K, p-AKT, and p-mTOR in a PD mouse model by protein blotting and also suggested BDNF inhibited apoptosis induced by MPP+ and cell proliferation, which was inhibited by siSTAT3 by CCK-8 and flow cytometry. BDNF enhances the phosphorylation of STAT3, which then activates the PI3K/AKT/mTOR signaling axis, regulating neuronal autophagy, preventing apoptosis, and encouraging cell growth. Meanwhile, their study also found that the BDNF group exhibited a notably decreased concentration of p-α-syn protein when compared to the MPP+ group, suggesting that BDNF protects neuronal cells by boosting autophagy and reducing the expression levels of α-syn. In this way, BDNF attenuated cell injury in the PD model. In astrocytes, BDNF-mediated activation of Nrf2 controls circadian rhythms, safeguarding dopaminergic neurons against ferroptosis (Ishii et al., 2019), thereby reducing the degenerative effects that PD may induce. BDNF also has a part in protecting against dopaminergic neurodegeneration in PD by inhibiting neuroinflammation and apoptosis, mainly targeting the pathways of NRF2 and NF-κB signaling interactions (Thirupathi et al., 2024). The potential value of BDNF as a treatment for PD is irrefutable, but it has also been shown that neither the direct delivery of peripheral BDNF to the brain nor promotion of the expression of BDNF gene alleviates the symptoms of PD, and that only physical activity that boosts the levels of BDNF in the brain is effective (Palasz et al., 2020). Although there have been many studies on the mechanism of BDNF in PD (Figure 2), we still need further research to explore the intrinsic mechanisms by which exercise promotes BDNF.

### 3.2 GDNF

GDNF is a homodimer that is glycosylated and contains disulfide bonds (Lin et al., 1993). It was initially extracted from cultured B49 rat glial cells (Lin et al., 1994). GDNF is distributed in both PNS and CNS, synthesized and released by various cells, including astrocytes, oligodendrocytes, and Schwann cells, and other multiple glial cell types (Sariola and Saarma, 2003).

Nowadays, there is sufficient evidence that exercise promotes GDNF production. BDNF and GDNF are elevated in the flounder muscle, after performing a round of exercise (Dupont-Versteegden et al., 2004; Koeda et al., 2024). Elevated levels of GDNF have been observed during treadmill training (Côté et al., 2011). Voluntary running triggers endogenous GDNF expression (Bonafina et al., 2019). Meanwhile, researchers demonstrated that swimming or running for two weeks changed the GDNF protein levels found in the lumbar spine and muscle tissues of rats that are young and old (de Azambuja et al., 2018; Gyorkos et al., 2014). Regarding the influence of different physical activity modalities on the promotion of GDNF, it has been demonstrated that various training modalities in young animals upregulate GDNF expression, but there are no notable distinctions between exercise modalities (McCullough et al., 2013). Physical training, which takes place both at the beginning and in subsequent stages, rescued nearly all dopaminergic neurons in the substantia nigra and ventral tegmental area, inhibited inflammatory responses, and raised levels of BDNF and GDNF to a comparable degree (Palasz et al., 2019). This could mean that exercise increases both GDNF and BDNF in PD and may have a combined effect. In Rabelo et al.’s (2017) study, the HP (high performance) group ran for 129 min per day, while the SP (standard performance) and LP (low performance) groups ran for 64 and 29 min per day, and although GDNF was elevated in all three groups, GDNF was significantly elevated in the LP group in comparison to the other two groups. This suggests that moderate training may be more effective than high-intensity training in promoting GDNF levels, although the optimal exercise intensity for enhancing GDNF remains to be determined.

**FIGURE 3:**
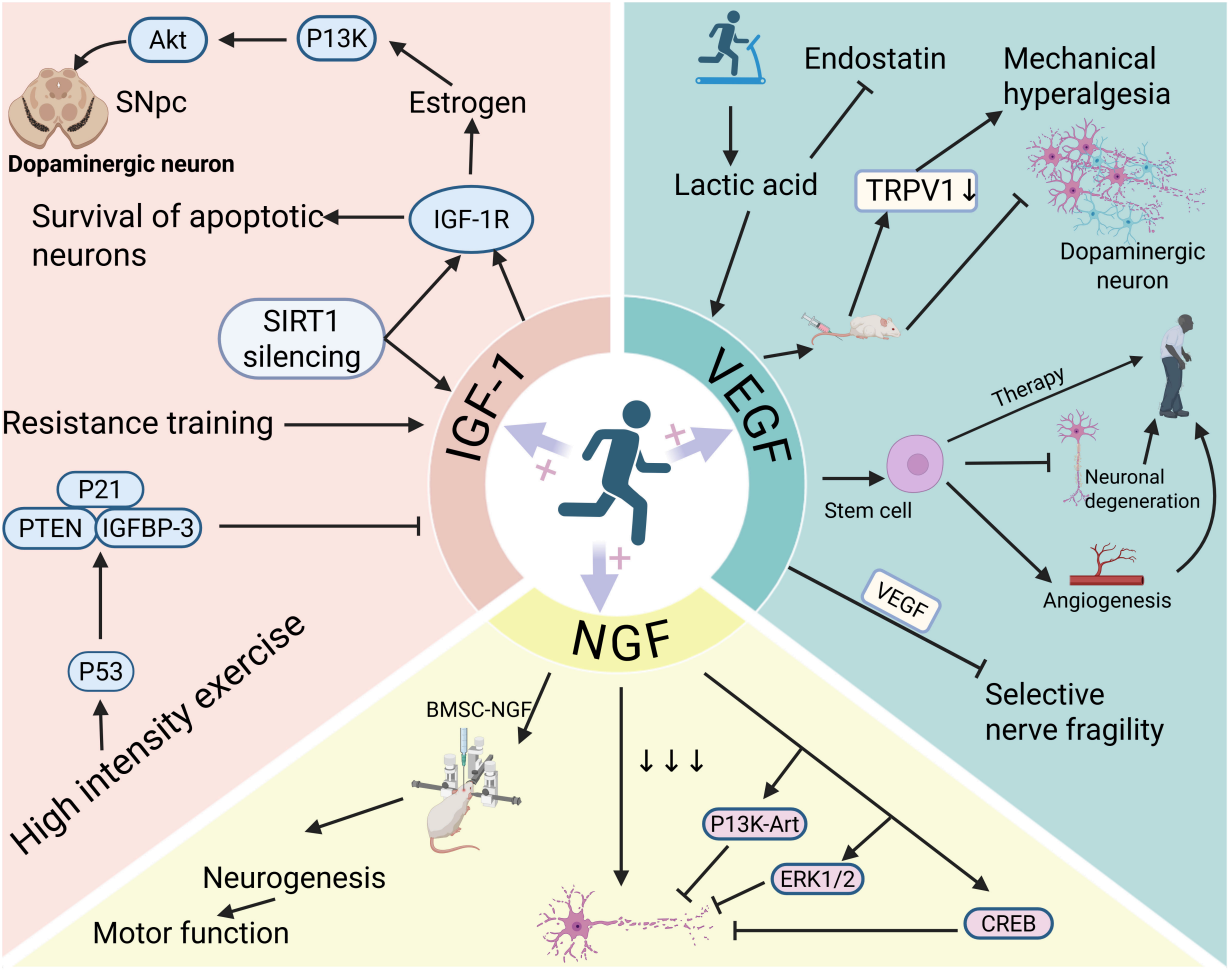
Exercise promotes growth factors for better treatment of PD. Exercise increases growth factors—IGF-1, VEGF, NGF. The elevated IGF level, after binding to IGF-1R, promotes the protection of dopaminergic neurons in the SNpc through the PI3K/Akt pathway by estrogen. VEGF and NGF through bone marrow derived stroma cell (BMSC), cAMP responsive element-binding protein (CREB) and other pathway receptors, promotes the survival of dopaminergic neurons and inhibits the progression of neurodegenerative diseases.

GDNF is a promising and significant therapeutic factor for PD (Figure 3). Studies indicate that dopaminergic neurons in primary cultures undergo survival and differentiation processes. GDNF improved these processes by promoting dopamine uptake and enhancing the capacity for high-affinity dopamine absorption, thus enhancing midbrain dopaminergic neurons’ ability to survive in mammals (Lin et al., 1993). The decrease of GDNF in the prefrontal cortex leads to synaptic connections and neural circuit degeneration, contributing to cognitive impairment in PD patients (Tang C. et al., 2023). In a 6-hydroxydopamine model of PD, forced exercise reduces the destruction of dopamine neurons by 6-hydroxydopamine, and further studies have found that this protective effect is partially due to increased availability of GDNF (Smith and Zigmond, 2003). Utilizing novel nanostructured delivery systems to increase GDNF expression can alleviate some cell-related symptoms related to PD observed in laboratory settings (Guzmán-Sastoque et al., 2024). The application of GDNF promotes better connectivity between transplanted dopamine neurons and the striatum and other targets typically supplied by natural dopamine neurons, thereby increasing dopamine levels in the striatum and restoring motor function (Moriarty et al., 2022). One team investigated the effects GDNF has on the survival of dopaminergic neuron and the transmission of dopamine at their terminals, revealing that a lack of GDNF negatively affects dopamine transmission in the prefrontal cortex (Tang C. X. et al., 2023). Moreover, that the dopaminergic protective effects it promotes are associated with responsive regulation of microglia. However, endogenous GDNF is not sufficient to protect dopaminergic neurons from inflammatory damage (Mendes-Oliveira et al., 2023). GDNF protects, restores, and enhances dopaminergic function of the pathway connecting nigra to striatum in a short-term animal model of PD (Mätlik et al., 2022). *RET* (rearranged in transfection) was identified as an oncogene activated by the recombination of DNA (Takahashi et al., 1985). *RET* is indispensable for the development of both the peripheral and central nervous systems (Mahato and Sidorova, 2020). Nurr1 is an important transcription factor regulating *RET* expression. Although accumulation of α-synuclein leads to down-regulation of Nurr1 and consequent attenuation of *RET* signaling, GDNF/NRTN therapy has the potential to restore this pathway through an “augmentation mechanism,” especially in neurons that retain some *RET* expression and thus up-regulate *RET* expression (Björklund, 2021). At the same time, GDNF/RET signaling may be a factor in Akt activation via a Src-dependent non-classical pathway that degrades α-nucleosynaptic proteins (Chmielarz et al., 2020).

Research shows GDNF-transfected macrophages effectively treat Parkin Q311X(A) transgenic mice which are characterized by slow progression and mild cerebral inflammation (Zhao et al., 2019). In late stages, they restore motor function and preserve brain tissue. Early injection in PD mice offers long-lasting benefits, reducing inflammation, α-synuclein buildup, and improving neuron survival and motor function. Thus, they’re promising for advanced and early PD. In conclusion, GDNF-transfected macrophages represent a promising therapeutic strategy for advanced diseases, including the early onset of PD (Zhao et al., 2019). In various clinical trials involving GDNF, PET imaging had indicated an increase in 18F-DOPA uptake. And there was no notable enhancement in the motor function of participants in placebo-controlled studies, which may be due to a lack of improvement attributed to the ineffectiveness of GDNF in enhancing the condition of damaged dopamine neurons (Barker et al., 2020), possibly suggesting that GDNF acts mainly during the initial phases of PD or primarily makes a preventative contribution. However, more GDNF is not necessarily better. Overexpression of GDNF can downregulate levels of tyrosine hydroxylase (TH), a key enzyme in dopamine synthesis, in rats, and may lead to abnormal sprouting and ectopic formation of synapses in the brain, resulting in various dose-dependent adverse effects (Georgievska et al., 2004).

## 4 Growth factors

Growth factors are a class of cytokines with the ability to stimulate cell growth. Most of them are proteins or polypeptides that can regulate key cellular activities such as growth, proliferation, and differentiation, playing crucial roles in human development, tissue repair, and immune regulation (Wang, 2021). Growth factors can protect and restore degenerated neurons while enhancing their functional activity, thus holding considerable promise for improving neurodegenerative diseases (Sidorova and Saarma, 2020). Exercise therapy can promote the production of growth factors, thereby facilitating the prevention and treatment of Parkinson’s disease (Pahlavani, 2023). Therefore, in this section, we will focus on discussing the impact of exercise on the production of IGF-1, VEGF, and NGF, as well as how these three growth factors exert neuroprotective effects.

### 4.1 IGF-1

A hormone called insulin-like growth factor 1 (IGF-1) is released by the liver cells, with its production increasing in the presence of growth hormone. IGF-1 stimulates myelination, oligodendrocyte maturation, neurite outgrowth, and neuronal survival in the brain (D’Ercole et al., 1996).

The effects of exercise on IGF-1 vary under different conditions, including the exercise population and type. Resistance training (RT), aerobic training (AT), or combination training (CT) raised IGF-1 levels in elderly individuals suffering from sarcopenic obesity, according to an experiment that looked at the effects of various forms of exercise on body composition, muscle strength, and IGF-1 levels. However, the effects of different exercise modalities on IGF-1 levels varied, with resistance training showing the most significant effect overall (Chen et al., 2017; Feng et al., 2022). This finding is also supported by other studies. The effects of several training modalities has been described on skeletal muscle mass, function, and IGF-1 signaling in aged mice in their research, Li et al. (2022) found that resistance training was superior to other forms of exercise in raising IGF-1 levels. Exercise has different impacts on IGF-1 levels in the short and long term. Stein et al. found that following acute resistance exercise, Alzheimer’s disease (AD) patients exhibited higher circulating IGF-1 levels compared to healthy individuals (Stein et al., 2021). Additionally, the duration and intensity of exercise significantly affect IGF-1 levels. One study discovered that after a single 30-min exercise session, IGF-1 levels in healthy older males increased; however, after 12 weeks of training, IGF-1 levels decreased (Behrendt et al., 2021). In a study of 14 healthy middle-aged males who participated in a 100-kilometer walking marathon, researchers observed a decrease in IGF-1 levels following high-intensity walking (Kim T. et al., 2015). This phenomenon is not only observed in humans but also animal models. In an experiment involving 20 male Wistar rats to investigate IGF-1 gene expression in colon mucosa with varying exercise levels, Buehlmeyer et al. (2007) found that serum IGF-1 levels decreased as exercise intensity increased. This reduction may be due to high-intensity exercise activating p53, leading to increased expression of p21, IGFBP-3, and PTEN, which induces negative regulation of the IGF-1 pathway (Yu et al., 2016). Notably, teenagers’ resting blood IGF-1 levels throughout brain development are positively impacted by prolonged, consistent aerobic activity (Jeon and Ha, 2015). Therefore, selecting an appropriately regular exercise regimen is essential to enhance serum IGF-1 levels while avoiding reductions associated with excessive exercise.

Male sex was found to be the highest-ranking risk factor, closely followed by serum IGF-1, when the relative impacts of non-genetic variables in Parkinson’s patients were examined using a comprehensive machine learning algorithm (IDEARS) (Allwright et al., 2023). Serum levels of IGF-1 were higher in Parkinson’s patients than in the normal group, with early-stage patients showing higher levels than those in mid-to late-stage disease, according to enzyme-linked immunosorbent assays conducted on serum IGF-1 levels in 100 healthy controls and 100 PD patients (including 49 early-stage and 51 mid- to late-stage cases). This may indicate that IGF-1 acts as a neuroprotective factor, compensatorily elevating in early PD to protect dopaminergic neurons from degeneration (Godau et al., 2010). Furthermore, in patients with PD, non-motor symptoms like anxiety, depression, and cognitive dysfunction were substantially inversely connected with serum IGF levels (Shi et al., 2023). Silencing information regulator 2 related enzyme 1 (sirtuin1, SIRT1) is an NAD-dependent deacetylase, which participates in a multitude of biological processes, including cell cycle regulation, DNA repair, apoptosis and inflammation, autophagy, and aging, and plays a pivotal role in preventing neurodegenerative diseases (Chen C. et al., 2020; Lamichane et al., 2019; Xie et al., 2019). SIRT1 silencing extended the survival of neurons with apoptotic damage by inducing IGF-1 protein expression and secretion, as well as increasing IGF-1 receptor (IGF-1R) protein levels (Sansone et al., 2013). Blocking central IGF-1 receptors (IGF-1R) can weaken estrogen’s neuroprotective effects on SNpc dopaminergic neurons. Additionally, the PI3K/Akt signaling pathway, not the MAPK/ERK pathway, is required to safeguard SNpc dopaminergic neurons by both estrogen and IGF-1 (Quesada et al., 2008). In a rat model of PD, human neural progenitor cells that overexpressed IGF-1 also preserved dopaminergic neurons and recovered their functionality (Ebert et al., 2008). The protective effect of IGF-1 on PD is undeniable (Figure 3), and it may also serve as an early biomarker for PD.

### 4.2 VEGF

Vascular endothelial growth factors (VEGFs) are members of the VEGF/PDGF (platelet-derived growth factor) group, which is a subfamily of the cystine knot superfamily of hormones and extracellular signaling molecules (Vitt et al., 2001), and are classified as secretory polypeptides. As a component of the PNS, the enteric nervous system (ENS) functions primarily independently while maintaining a connection to the CNS through the gut-brain axis. Hecking et al. (2022) provided experimental evidence of VEGF’s direct neuroprotective effects in the ENS. VEGF plays a significant and potent neuroprotective role following various types of central nervous system damage, such as epilepsy (Nicoletti et al., 2008), cerebral ischemia (Guo et al., 2016), oxidative stress (Cabezas et al., 2019), and neurodegenerative diseases (Guo et al., 2019; Lange et al., 2016). Calvo et al. (2022) found that a lack of retrograde transport of VEGF from the periphery affects the physiology of intact motoneurons, leading to a state akin to axotomy. This implies that VEGF is a necessary retrograde factor for motoneurons’ synaptic drive and discharge activity.

Exercise’s impact on VEGF has been the subject of numerous research studies. One study demonstrated that after eight weeks of moderate-intensity running, VEGF mRNA expression in rat skeletal muscle increased (Shin et al., 2015). Exercise raises VEGF levels through lactate generation, as evidenced by the fact that high-intensity interval training has been demonstrated to enhance VEGF-A levels in the brains of wild-type rats but not HCAR1 knockout mice (Morland et al., 2017). Zang et al. (2023) stated that exercise enhances the expression of the proangiogenic factor VEGF while reducing the expression of the antiangiogenic factor endostatin. This regulation occurs through a partially nitric oxide (NO)-dependent mechanism, promoting capillary growth in the cerebral cortex and improving cognitive function. Different types of exercise have different impacts on VEGF levels. It is noteworthy that VEGF promotes abnormal angiogenesis; therefore, anti-VEGF therapy is necessary in tumors and ocular diseases (Mabeta and Steenkamp, 2022; Mettu et al., 2021). Meanwhile, VEGF serves as a crucial neurotrophic factor for motor neurons, holding significant therapeutic potential in treating motor neuron diseases (Calvo et al., 2024). This underscores the context-dependent nature of VEGF’s effects, which cannot be reduced to a simplistic binary of “beneficial” or “harmful” but rather depends on its site of action, concentration levels, and surrounding microenvironment. Notably, exercise-induced VEGF upregulation typically represents a physiological, controlled, and mild elevation. At the conclusion of exercise, VEGF is increased by 128% ± 36% from resting levels when sprint interval training (SIT) is performed (*p* = 0.017). Conversely, other forms of exercise, like continuous moderate-intensity exercise and high-intensity interval training based on guidelines, do not exhibit comparable noteworthy gains (Weaver et al., 2021). Research indicates that acute endurance exercise elevates VEGF expression more intensely than strength training, while long-term endurance training enhances an individual’s sensitivity to VEGF responses during any exercise. Therefore, the exercise pattern and endurance training status significantly differ in their effects and mechanisms for increasing VEGF expression (Bizjak et al., 2021). Three hours post-exercise, long sprint interval training (LSI) and moderate-intensity continuous training (MIC) show significantly higher VEGF mRNA increases compared to short-duration high-intensity sprints (SSI), indicating that prolonged high-intensity interval training and moderate continuous training are more effective at activating VEGF gene expression than brief high-intensity sprints (Fiorenza et al., 2020). These results imply that exercise promotes VEGF production, regulates the balance of angiogenesis in the brain, and enhances vascular expansion (Soori et al., 2022).

VEGF supports the neuroprotection of dopaminergic neurons through both indirect and direct mechanisms, inhibiting the apoptosis of dopaminergic neurons *in vitro* (Mihci et al., 2011). In a mouse model of 6-hydroxydopamine (6-OHDA) damage, the injection of mesenchymal stem cells expressing VEGF189 significantly alleviates mechanical allodynia in PD mice, achieved by downregulating TRPV1 in the spinal dorsal horn (Li et al., 2023). VEGF treatment dramatically alleviated motor function impairments caused by 6-OHDA and prevented loss of dopaminergic neurons in the pars compacta of the substantia nigra (SNpc) and dopaminergic fibers in the striatum, according to another study that used a unidirectional 6-OHDA PD model. Furthermore, in the rat PD model, VEGF gene delivery inhibited microglial activation and apoptosis (Sheikh et al., 2017). In experiments involving stem cell therapy for PD, VEGF promotes local angiogenesis in brain tissue, potentially enhancing the survival rate of transplanted cells, and may also directly protect dopaminergic neurons, reducing their degeneration (Daviaud et al., 2015). Furthermore, there is a synergistic effect between VEGF and GDNF, aiding in the restoration of the dopaminergic system’s topological distribution in severe PD models and alleviating the selective neuronal vulnerability induced by 6-OHDA (Requejo et al., 2017). However, some teams suggest that oligomeric α-synuclein mediates the role of astrocyte-derived VEGFA in the disruption of the BBB associated with PD (Lan et al., 2022). Thus, the connection between PD and VEGF cannot be simply categorized as beneficial or harmful (Figure 3); its effects may vary at different sites or stages of PD progression, warranting further investigation.

### 4.3 NGF

Levi-Montalcini (1987) discovered and examined nerve growth factor (NGF), the first member of the neurotrophin (NT) family, at the beginning of the 1950s. NGF is a protein with a key role in the growth and differentiation of immature sympathetic nerve cells and in the maintenance of fully differentiated sympathetic neurons (Calissano and Levi-Montalcini, 1979). The cortex, hippocampus, and pituitary gland produce the most NGF, while the basal ganglia, thalamus, spinal cord, and retina also produce sizable amounts (McAllister, 2001). NGF is strongly related to the management of PD and is a crucial regulator of neuronal survival, development, and function. Its roles include regulating neuronal growth, proliferation, activation, and survival (Maes et al., 2023), modulating perception (Testa et al., 2021), maintaining cholinergic neurons (Bruno and Cuello, 2006), and influencing synaptic plasticity in limbic regions, thereby affecting learning and memory capabilities (Ciafrè et al., 2020). However, the mechanisms by which NGF elevation during exercise improves PD remain to be further explored.

Research indicates that NGF levels significantly increase following unilateral eccentric exercise, potentially linked to the onset of delayed onset muscle soreness (DOMS) (Koeda et al., 2024). Progressive exercise also notably raises NGF levels in the PD rats’ striatum (Fallah Mohammadi et al., 2019). After eight weeks of running training in rodents, NGF and its receptor, tropomyosin receptor kinase A (TrkA), are upregulated in the hippocampus (Hong et al., 2015). Cyclic AMP response element-binding protein (CREB) is activated when NGF binds to TrkA, promoting neuroplasticity and cell survival (Lin et al., 2018). Regular resistance training effectively reverses age-related declines in NGF levels (Rahmati et al., 2022). A study found that after four weeks of exercise, proNGF levels in the saliva of both adults and children decreased; particularly, this decrease was linked to increases in creativity and cognitive flexibility (Venditti et al., 2015), which may be because NGF is consumed after it is produced.

NGF is essential for neuron survival and differentiation and has garnered significant attention in neurodegenerative disease research (Figure 3). For instance, PC12 cells differentiated by NGF exhibit enhanced neurotransmitter secretion levels, reduced growth rates, and increased dendritic number and length closely resembling neurons. Thus, PC12 cells are considered an ideal cell line for modeling PD (Zhong et al., 2024). In experimental PD animal models, transgenic bone marrow stromal cells (BMSC-NGF) that produce NGF can be injected into the striatum to promote neurogenesis and markedly enhance motor capabilities (Wang et al., 2008). A reduction in NGF levels is commonly observed in PD, while PI3K/Akt, ERK1/2, and CREB signaling pathway activation may counteract this decline, thereby rescuing damaged neurons (Luo et al., 2018). Additionally, the intracerebral injection of AAV2-NGF has shown good tolerance and therapeutic effects on cognitive decline (Chen W. et al., 2020).

Many studies have shown that exercise significantly affects neurotrophic factors and growth factors (Table 2). However, there is currently no conclusive evidence indicating whether a synergistic interaction exists between the two in this process, which warrants further research.

**TABLE 2:** Comparative analysis of exercise interventions and changes in neurotrophic factor and growth factor levels.

References	Model	Exercise intervention	Exercise duration and frequency	Sample type and changes	Detection method
Tait et al., 2024	Older adults	Dual task functional power training (DT-FPT)	6 months, 45–60 min/time, 2 times/week	Blood, BDNF ↓ (*p* = 0.774), IGF-1 ↓ (*p* = 0.434) VEGF ↓ (*p* = 0.816) Compared with pre-exercise	ELISA (BDNF, VEGF), chemiluminescence immunoassay (IGF-1)
Kong et al., 2024	Members of the fitness center and community residents	Aerobic exercise	> 4 times/week, 2 h/time	Blood, BDNF ↑ (*p* = 0.014), GDNF ↑ (*p* = 0.027) Compared with the control	ELISA
Moghadasi et al., 2024	Sprague–Dawley rats	Aerobic exercise	8 weeks, 15–20 m/min, 15–30 min/session, 3 sessions/week	Hippocampus, BDNF NGF ↑ (*p* = 0.001) Compared with the control	qPCR
Rahmati et al., 2022	Wistar rats	Resistance training	17 session/5 weeks, 5 sets or 4 reps/time	Hippocampus, NGF ↑ (*p* = 0.006), BDNF ↑ (*p* ≥ 0.05) Compared with the control	Western blot
Tian et al., 2018	Sprague–Dawley rats	Swimming training	4 weeks, 60 min/time, 5 times/week	Ipsilateral spinal cord, NGF ↑ (*P* < 0.01) BDNF ↑ (*p* < 0.01) Compared with the control	Western blot

## 5 Inflammatory factor

The pathological characteristics and indications for PD are significantly influenced by inflammation. PD subjects’ brains, cerebral spinal fluid (CSF), and blood are known to contain elevated levels of a variety of inflammatory chemicals (Chen et al., 2018; Qin et al., 2016). Tumor necrosis factor (TNF) and interleukins are two examples of inflammatory indicators that are important signaling molecules of immunological activation that affect the peripheral systems and the brain (Tabas and Glass, 2013). While certain cytokines, chemokines, and other inflammatory indicators in PD patients’ peripheral blood exhibit irregularities, dysregulated variables such as IL-6, TNF, IL-2, IL-10, CRP, and CCL5 are frequently found (Qin et al., 2016; Qu et al., 2023), reinforcing the evidence of inflammation-related clinical manifestations in PD. Some scholars have proposed that under pathological conditions, the blood-brain barrier undergoes alterations, allowing the infiltration of peripheral immune cells and inflammatory factors. This leads to a reduction in neuroplasticity-related molecules such as BDNF and GDNF, further weakening the adaptive capacity of the central nervous system (Policastro et al., 2020; Patterson, 2015).

Exercise interventions show promise in enhancing brain plasticity and reducing neuroinflammation (Gupta et al., 2024; Liu Y. et al., 2024). Pro-inflammatory cytokines linked to low-grade chronic inflammation, including IL-6, TNF-α, and monocyte chemoattractant protein-1 (MCP-1), have been shown to be reduced by regular exercise (Martínez-Guardado et al., 2022). Exercise on a rotarod successfully reduced microglial activation and the expression of pro-inflammatory markers such as p-IκBα, iNOS, TNF-α, and cathepsin D in an MPTP mouse model, while raising the expression of anti-inflammatory IL-10 and TGF-β (Leem et al., 2023). Exercise significantly ameliorates inflammatory factors, especially IL-6, and TNF-α (Zhang S. et al., 2024). There are also a large number of experiments to prove this idea (Table 3). Therefore, we will focus on introducing these two inflammation-related factors: IL-6, and TNF-α.

**TABLE 3:** Comparative analysis of exercise interventions and changes in inflammatory factor levels.

References	Model	Exercise intervention	Exercise duration and frequency	Sample type and changes	Detection method
Malczynska-Sims et al., 2022	Parkinson’s disease patients	High-intensity interval training	12 weeks, 3 times/week	Blood, TNF-α↓ (*p* = 0.034) Compared with the control	ELISA
Vints et al., 2024	Older adults	Progressive resistance exercise	12 weeks, 2 times/week	Blood, IL-6 ↑ (*p* = 0.089) Compared with the control	ELISA
Bahmani et al., 2022	Multiple sclerosis patients	Home-based aerobic training	8 weeks, 3 times/week	Blood, TNF-α, IL-6 ↓ (*p* < 0.05) Compared with the control	ELISA
Wagner et al., 2015	Healthy young adults	Intense aerobic exercise	6 weeks, 3 times/week	Blood, TNF-α↑ (*P* < 0.009), IL-6, - Compared with the control	ELISA
Dunleavy et al., 2024	Human	Moderate-to-vigorous physical activity	6 weeks, 2–3 times/week	Blood, IL-6 ↓ (*p* = 0.006) Compared with the control	ELISA
Zoladz et al., 2014	Parkinson’s disease patients	Moderate-intensity interval training	8 weeks, 3 times/week	Blood, TNF-α↓ (*P* = 0.03) Compared with pre-exercise	ELISA
Jeong et al., 2024	Human	Resistance exercise	4 weeks, 4 times/week	Blood, TNF-α↓ (*p* < 0.05) Compared with the control	ELISA
Wang Y. H. et al., 2022	Healthy adults	Aerobic exercise	Varies, 3 times/week	Blood, TNF-α↓	ELISA

### 5.1 TNF-α

Carswell et al. (1975) experimentally proved that a protein known as “tumor necrosis factor” (TNF) enters an animal’s bloodstream after lipopolysaccharide assault and reticuloendothelial system stimulation. Activated mononuclear macrophage cells produce the homotrimeric protein known as tumor necrosis factor alpha (TNF-α), which consists of 157 amino acids (Horiuchi et al., 2010). It was the first identified factor with anti-tumor activity (Moore et al., 1999) and is also recognized as a systemic inflammatory response mediator (Blum and Miller, 1998). When activated microglia signal through TNF-α in response to aging, injury, or neuroinflammation, a neurotoxic astrocyte phenotype known as A1, A1-like, or neuroinflammatory reactive astrocytes is induced. These reactive astrocytes suppress supportive functions and start releasing chemokines like CXCL10, complement components like C3, and neurotoxic factors. These effects may help attract immune cells into the CNS and promote inflammation by allowing them to pass through the BBB (Lawrence et al., 2023). Research has indicated that PD patients had higher levels of TNF-α (Liu et al., 2022), underscoring the importance of investigating the intrinsic mechanisms of TNF-α in PD.

**FIGURE 4:**
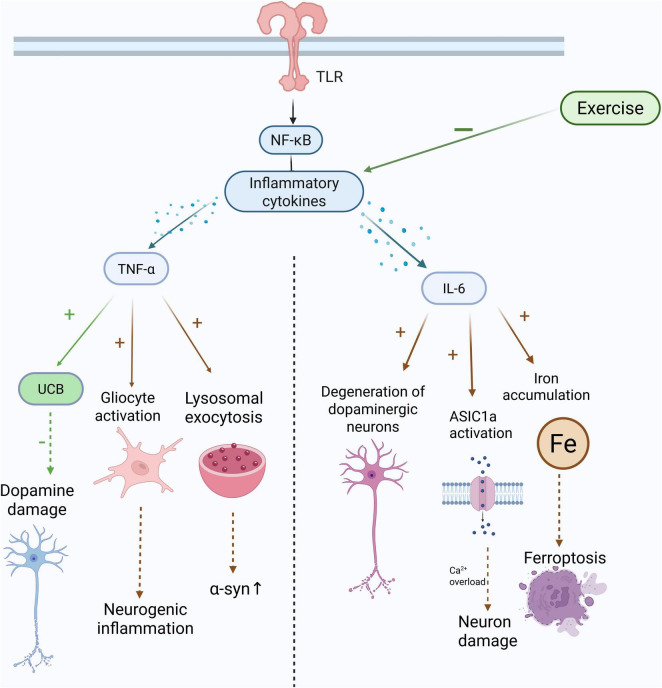
Exercise is anti-inflammatory and protects the brain. The Toll-like receptors (TLRs) on the surface of microglia and the nuclear factor kappa light-chain enhancer of activated B cells (NF-κB) is activated, thereby triggering the formation of inflammasomes and increasing inflammatory cytokines such as tumor necrosis factor (TNF), and interleukin-6 (IL-6). An experiment found that the elevation of IL-6 leads to dopaminergic degeneration. Exercise can reduce the neural damage caused by inflammatory factors and improve cognition.

The role of TNF-α in PD is dual-faceted (Figure 4). On one hand, TNF-α is a key mediator of neuroinflammation in PD, and its prolonged release can activate microglia and astrocytes, resulting in neurodegeneration and neuronal death (Amin et al., 2022; Shastri et al., 2013). In chronic PD macaque models, serum and CNS levels of TNF-γ and IFN-α were found to be consistently high, synergistically triggering glial activation within the substantia nigra (Barcia et al., 2011). On the other hand, TNF-α may exhibit neuroprotective properties under certain conditions, such as inducing unconjugated bilirubin (UCB) to counteract dopamine damage (Jayanti et al., 2022). Additionally, for the preclinical stage of PD, TNF-α is seen as a potential risk biomarker (Majbour et al., 2020), with plasma levels closely linked to elevated phosphorylated α-synuclein (p-α-syn), which is significantly connected to fatigue symptoms in PD patients, implying its possible involvement in distinguishing fatigued from non-fatigued PD patients (Wang et al., 2023). Interestingly, research in PD mouse models shows that gut microbiota can cause motor impairment by activating the TLR4/TNF-α signaling pathway, but fecal microbiota transplantation (FMT) can reduce symptoms by preventing neuroinflammation and TNF-α signaling (Sun et al., 2018). Moreover, TNF-α promotes the secretion of α-synuclein via lysosomal exocytosis (Bae et al., 2022) and exhibits complex interactions with α-synuclein fibrils during neuroinflammation; when both are present, TNF-α-induced pro-inflammatory responses are partially suppressed (Russ et al., 2021). In neurodegenerative illnesses, TNF-α plays a crucial role, as evidenced by the substantial correlation between high levels and the late course of PD (Piri et al., 2022).

### 5.2 IL-6

In 1973, interleukin-6 (IL-6) was first identified as a soluble factor secreted by T cells that is essential for antibody production by B cells (Hirano et al., 1986; Kishimoto and Ishizaka, 1973). IL-6 is a multifunctional cytokine that exerts both pro-inflammatory and anti-inflammatory effects (Scheller et al., 2011). IL-6 activates Janus kinases (JAK) by signaling either the trans-signaling pathway (sIL-6R) or the classical signaling pathway (mIL-6R) (Li et al., 2014), which in turn initiate three possible signaling pathways (Kaur et al., 2020). The first involves the phosphorylation of JAK’s own tyrosine, leading to the dimerization of STAT3 (Heinrich et al., 2003). The second causes MAPK to become hyperphosphorylated as well as exhibit increased serine/threonine kinase activity via activating the Ras/Raf pathway (Bousoik and Montazeri Aliabadi, 2018). The third activates the PI3K-PKB/Akt pathway, enhancing NF-κB activity (Ait-Ghezala et al., 2007). Increased levels of IL-6 are linked to neurodegenerative diseases (Shan et al., 2024), and research studies reveal that PD patients’ blood and CSF have higher levels of IL-6 (Liu et al., 2022).

Studies have shown that IL-6 plays a key regulatory role in the neurotoxicity of PD (Kozina et al., 2022). Peripheral IL-6 levels significantly influence the neurotoxicity induced by *LRRK2* mutations. For instance, in *LRRK2*-G2019S transgenic mice [where this mutation is a common pathogenic variant in familial Parkinson’s disease (Kozina et al., 2022; Omer et al., 2020), inhibiting IL-6 can alleviate the loss of nigral dopaminergic neurons induced by lipopolysaccharide (LPS) (Kozina et al., 2022)]. In the 6-hydroxydopamine (6-OHDA) model, overexpression of IL-6 exacerbates neuronal degeneration (Ma et al., 2020), suggesting a bidirectional interaction between *LRRK2* dysfunction and the IL-6 signaling pathway. In the mechanism, IL-6 enhances the activity of acid-sensing ion channel 1a (ASIC1a) channels, allowing more calcium ions to enter neurons, leading to calcium overload and neuronal damage (Castellanos et al., 2024). Increased IL-6 levels have been observed in carriers of *PRKN* and *PINK1* mutations, suggesting that both may act through a shared inflammatory pathway. This study provides the first evidence of elevated IL-6 in *PINK1* mutation carriers, supporting its role in inflammation associated with mitochondrial autophagy (Borsche et al., 2020). Additionally, levels of IL-6 is correlated with a reduction in gray matter volume in the left precentral gyrus and the worsening of motor complication scores (UPDRS-IV) in PD patients (Chen et al., 2024), suggesting that PD motor symptoms may be aggravated by structural changes in specific brain regions. Research by Sterling et al. (2022) highlighted IL-6’s key role in mediating neuronal iron accumulation in α-syn-related PD. Experiments in α-syn mutant mice and in models with α-syn pre-formed fibrils (PFF) revealed that IL-6 is essential for iron accumulation through trans-IL-6 signaling. Moreover, microglia drive iron accumulation in this process by secreting IL-6 and other factors. Ultimately, IL-6 overexpression leads to iron accumulation (Sterling et al., 2022). IL-6 secreted by astrocytes in PD can induce neuronal death through conditioned media, an effect that can be inhibited by the IL-6R blocking antibody tocilizumab, indicating the crucial role of IL-6 in neuroinflammation and neurodegeneration in PD (Pons-Espinal et al., 2024). This highlights the pivotal role of IL-6 in neuroinflammatory neurodegeneration and potentially extends its significance to *LRRK2*-associated PD. An analysis also indicated that the risk of PD is considerably decreased by IL-6 inhibitors (*P* < 0.001) (Fu et al., 2024). These evidence supports the IL-6 signaling pathway as a potential therapeutic target for *LRRK2*-associated and other hereditary PD subtypes. It is evident that IL-6 plays a central role in *LRRK2* mutation-mediated neurotoxicity and is intertwined with the pathways of hereditary and inflammatory PD. In summary, IL-6 plays an important role in the neuroinflammation of PD through various pathways (Figure 4). Targeting the IL-6 signaling pathway may offer neuroprotective strategies for different PD subtypes.

## 6 Conclusion and perspectives

### 6.1 Conclusion

Exercise exerts neuroprotective effects in PD by modulating multiple molecular factors. Myokines such as irisin and CTSB, secreted by skeletal muscles during physical activity, promote neurogenesis, mitochondrial function, and neuronal survival. Neurotrophic factors including BDNF, GDNF, and NT-3 support dopaminergic neuron maintenance and enhance synaptic plasticity. Furthermore, exercise downregulates pro-inflammatory cytokines such as IL-1β, IL-6, and TNF-α, alleviating neuroinflammation—a major contributor to PD progression. While it remains difficult to identify the most effective exercise type for regulating these factors, resistance training appears more effective in upregulating irisin, whereas aerobic exercise often enhances neurotrophic factor expression. Given the variability in individual response, exercise interventions should be tailored based on the patient’s physical status, preferences, and therapeutic goals to maximize benefits.

### 6.2 Limitations of existing research

Despite the widely recognized potential of exercise in treating PD, several limitations exist in current studies. First, detailed research on the specific molecular mechanisms of exercise, particularly how it mediates neuroprotective effects through factors like irisin, BDNF, and GDNF, is still lacking. Second, the effects of various forms of exercise on Parkinson’s patients are not entirely consistent and may even yield opposing effects in some cases. Moreover, individual differences such as disease progression, age, sex, and physical ability can significantly influence the effectiveness of exercise interventions. Most existing studies focus on specific populations, neglecting a comprehensive consideration of varying individual characteristics. Particularly for patients who have lost or have limited mobility, finding alternative therapeutic approaches (such as medications or physical therapy) that can simulate the neuroprotective effects of exercise remains a pressing issue. Furthermore, existing research predominantly addresses short-term exercise interventions, while not enough research has been done on the long-term consequences of exercising. Given that PD develops over time, symptoms and pathological changes worsen over time. Therefore, examining the long-term advantages of exercise and whether sustained exercise can continuously slow disease progression is a crucial avenue for further investigation.

### 6.3 The prospect of future research

Further exploration is needed regarding the relationship between cytokines and PD, particularly how various cytokines regulate neuronal plasticity and survival through specific signaling pathways. Additionally, investigating how to pharmacologically or otherwise simulate the effects of exercise-induced cytokines or enhance their activity within neurons may represent crucial strategies for patients unable to engage in exercise. Although the cytokines mentioned in this review are believed to be influenced by exercise and contribute positively to neuroprotection, it remains unclear whether they exhibit synergistic effects or act through different signaling pathways on dopaminergic neurons. Future research could further employ animal models or clinical trials to elucidate the mechanisms of these factors in PD patients and determine their possibility as targets for combined therapies. Furthermore, excessive exercise may induce oxidative stress, exacerbating neuroinflammation. Thus, understanding how to achieve a balance between anti-inflammatory and antioxidant systems during exercise will be an important focus. Specifically, research can investigate how different exercise intensities affect inflammatory cytokines to identify the optimal exercise dosage and minimize adverse reactions.

Individual differences among PD patients are significant; factors such as disease stage, physical constitution, and exercise ability may all influence treatment efficacy. Therefore, future studies should delve deeper into personalized exercise intervention programs to ensure that diverse patient groups might benefit from exercise. As the disease progresses, the severity of symptoms varies. For early-stage patients, exercise can serve as a preventive intervention to delay disease progression, whereas tailored exercise programs are needed for mid- to late-stage patients to address motor symptoms and alleviate non-motor symptoms. For example, mild patients may engage in moderate to high-intensity aerobic exercises to maximize neurotrophic factor secretion, while moderate to severe patients may benefit more from low-intensity rehabilitative training to avoid overburdening their bodies. Given the significant variability in exercise capacity among patients, establishing an individualized assessment system for exercise ability is essential to determining the appropriate intensity for each patient. Future research could develop assessment tools to evaluate various parameters such as cardiopulmonary function, muscle strength, and balance, thereby facilitating the creation of personalized exercise prescriptions. Different forms of exercise can specifically target various Parkinson’s symptoms. For instance, yoga and Tai Chi can enhance postural stability and flexibility; treadmill training can improve gait and coordination; and inspiratory muscle training can help alleviate dysphagia and other non-motor symptoms. Future studies can design targeted exercise programs based on the specific symptoms presented by patients to maximize the efficacy of exercise interventions.

In short, exercise has demonstrated promising effects in reducing the risk of developing PD and in alleviating its symptoms. However, the underlying molecular mechanisms through which exercise confers neuroprotective effects remain incompletely understood. Uncovering these mechanisms will require sustained, in-depth research efforts. A better understanding of these pathways may help integrate exercise more effectively into comprehensive treatment strategies, ultimately enabling more individuals with PD to benefit from its therapeutic potential.
